# Bone remodeling: an operational process ensuring survival and bone mechanical competence

**DOI:** 10.1038/s41413-022-00219-8

**Published:** 2022-07-18

**Authors:** Simona Bolamperti, Isabella Villa, Alessandro Rubinacci

**Affiliations:** Osteoporosis and Bone and Mineral Metabolism Unit, IRCCS San Raffaele Hospital, Via Olgettina 60, 20132 Milano, Italy

**Keywords:** Osteoporosis, Bone, Homeostasis

## Abstract

Bone remodeling replaces old and damaged bone with new bone through a sequence of cellular events occurring on the same surface without any change in bone shape. It was initially thought that the basic multicellular unit (BMU) responsible for bone remodeling consists of osteoclasts and osteoblasts functioning through a hierarchical sequence of events organized into distinct stages. However, recent discoveries have indicated that all bone cells participate in BMU formation by interacting both simultaneously and at different differentiation stages with their progenitors, other cells, and bone matrix constituents. Therefore, bone remodeling is currently considered a physiological outcome of continuous cellular operational processes optimized to confer a survival advantage. Bone remodeling defines the primary activities that BMUs need to perform to renew successfully bone structural units. Hence, this review summarizes the current understanding of bone remodeling and future research directions with the aim of providing a clinically relevant biological background with which to identify targets for therapeutic strategies in osteoporosis.

## Introduction

Despite the tremendous efforts of researchers studying bone remodeling for more than 50 years, the intrinsic spatial, biomolecular, and mechanotransduction complexities in bone remodeling continue to be debated. Initially, a two-stage process involving two types of cellular machinery was considered to be responsible for bone remodeling, in which bone formation by osteoblasts follows bone resorption by osteoclasts to achieve net bone mass equilibrium upon physiological maturity.^1^ However, recently identified cellular events, coordination pathways and anatomical structures have allowed a better understanding of the genesis, differentiation, activity, crosstalk and death of the entire cell population involved in bone remodeling.^2^ These discoveries have shed new light on the operational processes ensuring bone mass renewal without bone mass loss under physiological conditions. Bone remodeling is activated by local and systemic factors, supporting the concept that targeted remodeling is activated by local factors and that stochastic remodeling is activated by systemic factors, with these factors cooperating to maintain mechanical competence and meet concurrent metabolic demands. This new information implies that the determinants of the focal balance in bone mass after remodeling are the integrated effects of both mechanical and metabolic environmental conditions.

This review provides a novel integrated picture of the operational bone remodeling processes by describing, updating, and reexamining the current evidence and its biological plausibility. In this review, bone remodeling is described as a continuous flow of cellular signaling and connected events, not as a process comprising stages, as it has been historically presented.

## Bone modeling, remodeling, and the mutual regulation of bone resorption and formation

In vertebrates, bone modeling and remodeling are essential processes that are activated throughout life and are regulated by distinct temporal cellular constituents that ensure functional bone adaptation and vertebrate survival. Bone modeling adapts bone shape to variable mechanical demands during growth and aging through cellular events that determine bone resorption and formation on opposing cortical and cancellous surfaces. This implies the existence of a modeling drift, which moves a bone structural unit over time in the direction defined by growth patterns, and adjusts the bone mass distribution to the stresses and strains induced by locomotion and physical activity.^3^ By bone remodeling, old or damaged bone is replaced with new bone through a sequence of cellular events occurring on the same surface without any change in bone shape.^4^

In the 1960s, Frost^5^ recognized that a forming osteon in mammalian compact bone consists of a group of synchronous cells, suggesting “control mechanisms which are functionally and temporally ordered, discontinuous and discrete”. Frost advanced the enlightening notion of a basic multicellular unit (BMU) as a transient anatomic structure in bone remodeling and introduced the quantum concept of bone remodeling, which is analogous to *quantum* theory in physics. *Quantum* physics explains the property of matter at the smallest scale. It defines the behavior of the minimum, discrete amount, i.e., the quantum, of any physical entity on the assumption that all phenomena in a submicroscopic system exhibit quantization*.*^6^ In an analogy, Frost intuitively defined a quantum of bone remodeling as a discrete change in bone mass resulting from the coordinated activity of an individual BMU at an anatomically discrete locus.^1^ The quantum concept conceived by Frost and later extended by Parfitt^7^ has had profound implications for the understanding of all aspects of bone pharmacology and physiopathology,^8^ particularly in the osteoporotic context*.*^9^ A remarkable idea of the early sixties that is in line with the modern theory that quantum biological phenomena can lead to evolutionary advantages*.*^10^

**Fig. 1:**
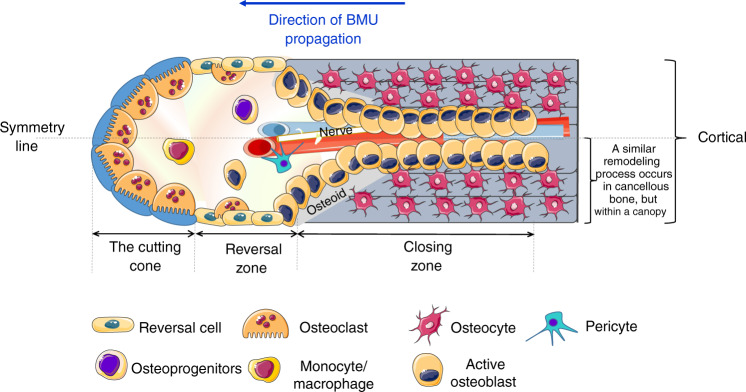
The cutting cone. The cutting cone originates in close proximity to neurovascular axial bundles and is generated by the propagation of the basic multicellular unit (BMU). The cutting cone consists of a set of osteoclasts, followed by a set of osteoblasts, reversal cells and secondary osteoclasts that cover the so-called reversal zone. At the end of the reversal zone, a set of osteocytes generate the closing zone. A line of symmetry divides in half the representation of a complete BMU in the cortex moving toward the longitudinal axis of the long bone. One-half of cortical BMU is similar to the BMU at the cancellous surface, although in cancellous bone, the BMU is separated from the marrow by a specific cell structure called the canopy

Frost recognized that the change in bone mass caused by the focal balance in each remodeling cycle of resorbed bone and formed bone is not an outcome of isolated “working” cell packets but is derived from the interacting parts of the whole BMU, which endures longer (9 months) than the lifespan of each single component.^4^ This implies a continuous and ordered cell supply that depends on the division frequency of each progenitor cell and the lifespan of each differentiated cell. A tightly maintained equilibrium between genesis and apoptosis is therefore critical for a properly functioning BMU.^11^ Recent advances have added complexity to the original BMU description. The number of cells considered to constitute a BMU has expanded to include all bone cells at all differentiation stages, interacting with their progenitors, T cells and bone matrix components. The BMU includes, in particular, a set of osteoclasts localized in the “cutting cone” followed by a set of cells, including reversal cells and osteoblasts, localized at the reversal zone; and a set of osteocytes localized in the closing zone. These cell sets constitute a secondary osteon around the neurovascular bundle wich is axially located with the connective matrix in the Haversian canal. A visualization of a complete BMU can be acquired only with a longitudinal section of cortical bone, where Haversian canals run parallel to the sectional plane (Fig. 1). Notably, the 3D trabecular network prevents the proper visualization of a complete BMU in cancellous bone.^12^

In bone remodeling, osteoblast–osteoclast interactions are necessary and must be coordinated in time and space to maintain the focal bone remodeling balance; this balance refers to the net amount of tissue resorbed and formed at each remodeling site (i.e., focal point) to maintain the structural integrity of the tissue. At maturity, the focal balance (neutral) in bone remodeling is determined by equal bone aliquots, which are removed and synthetized. In other periods in life or as a consequence of metabolic diseases, the focal balance can be negative (bone mass is lost) or positive (bone mass is gained). Focal coupling in bone remodeling strictly refers to the transfer of the information within the cell pool responsible for bone remodeling. This operational process couples the events necessary to remove and replace bone units under a hierarchy of time and space, independent of their focal balance; in fact, focal remodeling imbalance does not imply “uncoupling”. At each remodeling site, coupling indicates that the receptors on the osteoclast membrane as well as the regulatory factors released by osteoclasts are coupled with the sequential recruitment and differentiation of osteoblast lineage cells toward the mature phenotype, laying down the bone matrix and inducing mineralization. Coupling therefore implies that a commensurate change in bone formation follows any pathology- or therapy-related modification of bone resorption.

At each remodeling site, coupling is asynchronous: at any given time, bone is being resorbed at some sites, while it is being formed or is in the reversal phase from bone resorption to bone formation at other site. This implies that there is continuous focal transient loss of bone that is fully reversed in balanced remodeling; as the number of activated remodeling sites increases, the transient loss of bone, which is defined as the “virtual space” of bone remodeling, increases. As coupling takes place in different locations at different times, it requires local regulatory factors to transfer information among cells and tissue locations according to bone remodeling needs. Therefore, remodeling is defined as a dynamic physiological process executed through the coupled activity of osteoclasts and osteoblasts belonging to a BMU. Conversely, bone turnover is the outcome of bone remodeling that defines the amount of bone removed and formed within a given volume in a given time and is determined by the number of BMUs and by the focal balance within each BMU. As Parfitt said *“Bone turnover refers to proportional bone volume replacement per unit time, and is usually expressed as percent/year […]. If bone turnover is 10%/year, then the mean lifetime of each moiety of bone is 10 years”*.^13^

## Osteocytes: tuning, initiation and termination of bone remodeling

Osteocytes are a relatively permanent phenotype of bone cells that Frost estimated to have a lifespan of ~25 years^1^ and play a master role in bone remodeling. Osteocytes represent more than 90% of bone cells in the adult skeleton^14^ with a average cell density highly dependent on the specie, bone anatomical site, and subiect analyzed.^15^ Recent studies performed using new imaging techniques have estimated that 19 000–28 500 cells per mm^3^ populate the human skeleton, accounting for ~42 billion individual cells.^16^ Osteocytes consist of a stellate body connected by slender cell processes (50–100 processes per cell). The asymmetrical *“arborization”* of dendrites polarizes toward bone surfaces, where the dendrites contact osteoblasts undergoing growth or remodeling or, during bone surface quiescence, bone lining cells. The term “*arborization”* was originally introduced by Marotti and Palumbo,^17^ and it now refers to “dendrogenesis”, the asymmetric and asynchronous formation of osteocyte dendrites. Short radiating dendrites extend toward mineralized surface, and long radiating dendrites extend toward the vasculature during the progressive translocation of cell bodies farther from the vascular surface due to the secretion of the osteoblastic lamina. Osteocytogenesis and dendrogenesis are discussed in a subsequent section (“The osteoblast pool: recruitment, expansion and osteocytogenesis”).

An adult human skeleton includes 23 trillion osteocyte connections with each other and with bone surface cells.^16^ These connections form a 3D protoplasmic network that constitutes a matrix-integrated functional syncytium, which does not cross cement lines but does establish direct contact with the bone marrow, resides in low-oxygen microenvironment and comprises multiple elements. During aging in both humans and rodents, this network deteriorates. Throughout aging, a large and linear reduction in dendrite number and cell body density directly related to the deterioration of cortical parameters has been observed.^18^ Since decreased osteocyte number accompanies reduced dendritic density, it has been suggested that dendrite loss might contribute to diminished osteocyte viability because a certain degree of locally and/or systemically triggered anabolic signaling through the osteocyte-lacunocanalicular system is lost (see below).^18^

The osteocyte-bone lining cell syncytium displays gap junctions (connexins) that allow the transfer of information between cells. Connexin43 (Cx43) is the most abundant connexin in osteocytes, and global knockout of Cx43 expression is lethal at birth.^19^ Conditional knockout of Cx43 in osteoblasts and osteocytes in mice led to various degrees of osteopenia depending on the differentiation state in which the deletion was induced,^20,21^ suggesting that functional Cx43 in osteoblasts and osteocytes is essential for normal bone mass acquisition and maintenance.

The osteocyte-bone lining cell syncytium is endowed within the lacunocanalicular network of cavities filled with bone extracellular fluid (BEF). BEF has a different ionic composition from systemic extracellular fluid (SEF)^22^ and establishes an extensive contact surface (215 m^2^, which is 120-fold the size of the trabecular network) with the mineralized matrix to allow efficient, short-term mineral exchange with SEF.^23–26^ This specific physical environment allows osteocytes to govern metabolic demands and mechanotransduction for bone mass adaptation.^27^ The strain-induced flow of BEF in the lacuno-canalicular network exerts a shear stress (fluid flow shear stress, FFSS) on osteocyte bodies, which undergo dendrogenesis to activate several classes of mechano-sensors regulating specific gene expression patterns.^28^

Osteocytes might sense FFSS through 1. “collagen hillocks”, which are collagen matrix projections in osteocyte canaliculi that directly link the matrix to osteocyte dendrites;^29^ 2. β3 and β1 integrins, which participate to focal adhesion kinase (FAK) complex formation in osteocyte dendritic projections;^30^ 3. primary cilia, which have a flow-sensing function that leads to increased osteoprotegerin (OPG)/receptor activator of nuclear factor kappa-B ligand (RANKL) ratio via a calcium-independent mechanism;^31^ 4. connexin43, a component of gap junctions that mediates the transduction of mechanical signals;^32^ and 5. mechanosensitive ion channels, such as those composed of Piezo1, which are highly sensitive to osteocyte membrane tension.^33^

The deformation of the osteocyte cytoskeleton elicits Ca^2+^ influx signaling via the activation of TRPV4.^34^ The generation of Ca^2+^-dependent contractions of the cell membrane favors the production and release of extracellular vesicles (EVs) containing bone regulatory proteins.^35^ EVs are ubiquitous lipidic organelles that mediate the intercellular transfer of information through their cargo, which includes both proteins and nucleic acids.^36^ In osteocytes, EVs can be observed in proximity of the osteocytic network.^37^ In response to mechanical stimuli and subsequent Ca^2+^ influx, osteocyte lines have been observed to release EVs containing RANKL, OPG, and sclerostin.^35^

All the mechanical signals received by the osteocyte-bone lining cell syncytium modulate cell apoptosis and survival as well as the anabolic Wnt pathway in bone. Secreted Wnt protein stimulates target cell via the β-catenin-mediated (canonical) and β-catenin-independent (noncanonical) pathways. The canonical pathway has emerged as the predominant component of Wnt signaling in bone, positively affecting the entire osteoblast lineage.^38^ Wnt proteins bind to their receptors (Frizzled) and coreceptors (LRP5/6) to promote the stabilization of β-catenin in the cytoplasm, which translocates to the nucleus, where it induces the expression of osteogenesis-related genes.^38,39^ Many studies have demonstrated that β-catenin is required for bone formation and is activated during multiple stages of osteoblast differentiation to regulate both osteoblast and osteoclast*.*^40–43^ In fact, WNT–β-catenin signaling in osteoblasts and osteocytes indirectly represses osteoclast differentiation and bone resorption by stimulating the secretion of OPG.^38^

Mechanical signals activate the anabolic Wnt pathway in bone through the suppression of the Wnt receptor antagonist sclerostin (SOST). Recently, Sato et al.^20,44^ showed that FFSS induces the disruption of the FAK-integrin complex, thereby inhibiting histone deacetylase 4/5 (HDAC4/5), a negative regulator of SOST expression in osteocytes^45^ modulated by parathyroid hormone (PTH) treatment.^46^ Osteocytes can also decrease SOST expression after sensing decreases in oxygen levels.^47^

In vivo anatomical studies have suggested that the presence of apoptotic osteocytes at microdamaged sites correlates with the recruitment of osteoclasts at microcracks.^48^ In addition to their fundamental role as mechanosensors, osteocytes appear to be spatially, temporally and mechanistically linked to bone remodeling activation, particularly by regulating RANKL (as is discussed below). RANKL is routinely found as a membrane bound protein (mRANKL), but it can also be cleaved into a soluble form (sRANKL)^49^ or delivered via EVs.^35^ RANKL binds the receptor activator of nuclear factor kappa-B (RANK) on osteoclasts to induce osteoclastogenesis.^50^

In 2011, Nakashima et al.^51^ showed that the conditional deletion of RANKL expression in osteocytes using a DMP1 promoter caused a severe osteopetrotic phenotype. This osteopetrotic phenotype was confirmed by Xiong et al.,^52^ who in addition found that RANKL mRNA, isolated from total bone of DMP1-cre^+^, RANKL^loxp/loxP^ mice, showed very little variation compared to that of wild type mice. The concentration of circulating sRANKL was unaltered compared to that in wild type animals. However, a 70% reduction in osteoclast number in the cancellous bone of these transgenic mice highlighted the relevance of local RANKL production.^52^ As the activation of Dmp1-Cre promoters is not exclusive to osteocytes,^53^ the conditional deletion of RANKL expression was performed under the control of the more specific SOST promoter. This conditional deletion generated a phenotype resembling the previous one, with a significant decrease in osteoclasts number in cancellous bone.^54^ Taken together, these in vivo studies confirmed that osteocytes produce RANKL and that osteocytic RANKL is fundamental to sustaining bone remodeling.

Whether osteocyte RANKL is soluble, is transferred via vesicles or is membrane-bound is not fully clear. Bonewald et al.^14^ first demonstrated that the MLO-Y4 osteocyte line expresses RANKL at the cell surface and at dendritic processes. MLO-Y4 cells and primary murine osteocytes cocultured with bone marrow precursors supported osteoclastogenesis, but their conditioned media alone did not have a similar effect,^55^ suggesting that cell–cell interactions between osteocytes and osteoclasts are needed for osteoclastogenesis. In a different study, the conditioned media of apoptotic MLO-Y4 cells increased osteoclast formation, osteoclast size and osteoclast precursor migration. Interestingly, in apoptotic MLO-Y4 cells, both RANKL mRNA and protein expression were upregulated, suggesting that the conditioned media of this cell culture might contain sRANKL.^56^ On the other hand, the conditioned media of a non apoptotic MLO-Y4 culture did not alter any of these aforementioned cell parameters.^56^ In another in vitro study, primary osteocytes were cocultured on a 3D collagen scaffold with osteoclast precursors separated by a porous membrane. Again, the osteocytes supported osteoclast formation, but the efficiency of their effect decreased with a decrease in the pore size of the membrane. Confocal microscopy showed that large pore membranes allow osteocyte dendrites to touch osteoclast precursors through the pores, but this contact was not observed when small-pore membranes were used.^57^ Therefore, the authors of this study hypothesized that under specific conditions, such as when osteocytes cannot physically touch OC precursors, osteocytes might support osteoclastogenesis by secreting EVs containing RANKL*.*^58^ Distinguish the exact localization and form of RANKL produced by osteocytes in vivo is particularly complicated, as the antibodies used for immunohistochemical analyses do not distinguish between the soluble and membrane forms. Therefore, to evaluate the form of RANKL that induces osteoclastogenesis, a mouse model was generated with a sheddase-resistant form of RANKL with no detectable sRANKL levels in the circulation. During growth, this lack of sRANKL did not cause bone mass alteration, but in the adult model mice, the number of osteoclasts was reduced with an increase in cancellous bone mass.^59^ However, mice lacking sRANKL expression and ovariectomized still displayed bone loss because of the lack of estrogen, indicating that, although mRANKL can support most RANKL functions, the soluble form is necessary to sustain bone remodeling under certain conditions.^59^ In support of this hypothesis, mice overexpressing sRANKL only in the liver, and therefore also in blood circulation, displayed a significant decrease in bone mass with an increase in the number of osteoclasts on cancellous bone surfaces during aging.^60^

**Fig. 2:**
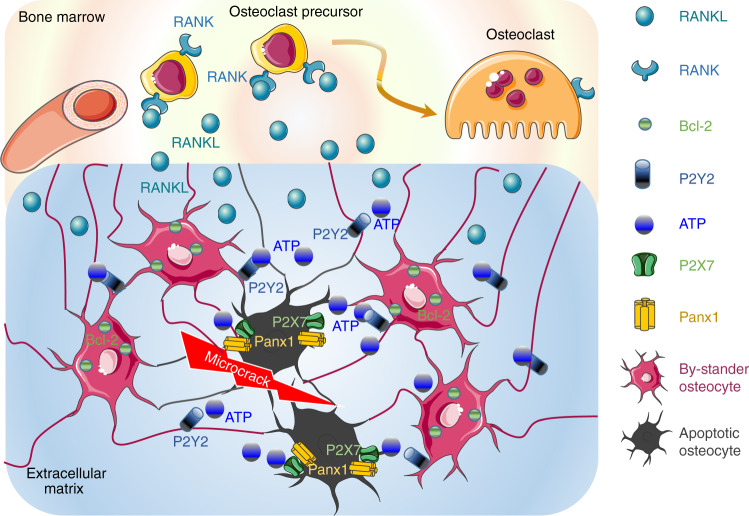
The “find me” message from apoptotic osteocytes. Under fatigue failure, osteocytes undergo apoptosis, whereas the osteocytes surrounding the apoptotic osteocytes, the “bystander osteocytes”, show upregulation of the antiapoptotic protein BcL-2, which protects them from death. Apoptotic osteocytes activate the Panx1/P2XR pathway to induce the release of ATP into the extracellular compartment as a specific “find me” signal. ATP binds P2Y2 receptors on bystander osteocytes, which in turn produce and release RANKL at the interface with the bone marrow. RANKL promotes the recruitment of osteoclast progenitors and osteoclastogenesis

In vivo, osteocyte apoptosis is fundamental for initiating bone remodeling,^61^ independent of the stimulus causing osteocyte death such as estrogen loss,^62^ fatigue,^63^ or unloading*.*^64^ An in vivo immunohistochemical analysis showed that the RANKL signal in bone tissue was stronger in an area around 150–200 μm from the apopototc osteocytes.^65^ Interestingly, inhibiting apoptosis prevented the RANKL production by neighboring osteocytes, suggesting that “bone remodeling follows a common paradigm for localized tissue repair”, with different response-induced factor released from apoptotic osteocytes near the damage site and “bystander” osteocytes.^63^ MLO-Y4 cell apoptosis generated ATP release from cells via Pannexin 1 channels (PANX1) opening.^66^ The same phenotype was observed in mice treated with an inhibitor of the P2X7 receptor (P2X7R), which is a coactivator of PANX1.^67^ In periodontal ligament cells, ATP induced the upregulation of RANKL expression via a P2Y1 receptor-cyclooxygenase-dependent pathway.^68^ Since the release of ATP from apoptotic cells functions as a specific “find me” message for phagocytic cells,^69^ ATP release during osteocyte apoptosis might be a specific “find me” signal directed to neighboring osteocytes. Indeed, ATP from apoptotic osteocytes might bind P2Y2 receptors expressed on bystander osteocytes, which in turn might activate RANKL production and release^67^ (Fig. 2). Moreover, bystander osteocytes (in 1–2-mm proximity to the focal damage site) expressed the antiapoptotic protein BcL-2. This defense mechanism might be necessary to prevent osteoclastogenic signal damaging viable osteocytes^70^ (Fig. 2).

As osteocytes are deeply embedded in the bone matrix, osteocyte apoptosis or autophagic death causes secondary necrosis due to inhibited phagocytosis by scavenger cells. Necrotic osteocytes release damage-associated molecular pattern (DAMP) proteins after the cell membrane is disrupted.^61^ DAMP proteins flow through the canalicular network and reach the bone/marrow interface, where they bind to pattern recognition receptors (PRRs) on bone marrow cells.^71^ When activated by DAMP–PRR binding, monocytes and macrophages produce proinflammatory cytokines, such as TNFα, IL-6, and IL-1*.*^72^ These cytokines in turn stimulate the expression of RANKL in osteoblasts,^73^ enhancing the osteoclastogenesis previously activated by the release of ATP by apoptotic osteocytes.^66^ DAMPs can directly regulate osteoclast formation by activating the membrane-bound C-type lectin receptor Mincle. Mincle activation triggers calcium signaling and oxidative phosphorylation in osteoclasts, inducing osteoclast differentiation.^74^

Under physiological conditions, osteocytes exert an inhibitory effect on osteoclasts by contributing to the osteoblast production of OPG via Wnt signaling.^75,38^ OPG functions as a soluble decoy receptor that binds RANKL, preventing its interaction with RANK expressed by osteoclast progenitors. The major cell source of OPG is still debated, as it depends on the bone compartment and on the age of the subject considered. In addition to osteocytes, other cells can be sources of OPG or RANKL; these cells are osteoprogenitors, osteoblasts^49^ or cells of the immune system (B and T cells),^76^ which are all simultaneously present in a BMU. Nevertheless, OPG released by mature osteoblasts and/or osteocytes may constitute the termination signal of the resorption phase in the remodeling cycle.^77^

In addition to microcrack-induced signals to activate the healing of microdamage foci through targeted remodeling, osteocytes sense systemic and local stimuli that affect their functions and survival. When exposed to a stimulus, osteocytes release several factors affecting (1) bone remodeling at stochastic loci, (2) matrix mineralization, (3) lacunocanalicular remodeling, (4) mineral homeostasis, and (5) fat metabolism and myogenesis (Table 1).

**Table 1:** Systemic and local factors influencing osteocyte function

Stimuli (Systemic/Local)	Features affected (Local)	Factors released (Local/Systemic)	Final outcome (Systemic/Local)
PTH Estrogens 1,25(OH)_2_D_3_ Irisin Calcitonin Senescence TGF-β1 Pi pO_2_	Life-Span Network formation Secretome Mechanotrasduction	Sclerostin, DKK1, FGF23, DMP1, Phex, MEPE, Sema 3A RANKL, OPG SASPs, TGF-β1 OCN	• Bone Remodeling Balance • Matrix Mineralization • Perilacunocanalicular Remodeling • Errors correction in Ca^2+^/P homeostasis • Energy metabolism • Myogenesis • Oxidative stress

Major systemic and local stimuli that influence osteocyte activity, the functions affected, the factors released upon exposure to the stimuli, and the impact on the system

### PTH/PTH-related peptide (PTHrP)

Under physiological conditions, osteocytes integrate the responses of bone to mechanical loading and PTH, since the mechanotransduction process requires PTH receptor 1 (PTHR1) activation to downregulate SOST expression. SOST expression downregulation positively fine-tunes the bone remodeling balance by promoting osteoblast expansion.^78^ When PTHR1 on osteocytes is activated by chronically high PTH concentrations or when PTHR1 is constitutively active, osteocytes initiate bone remodeling by expanding the osteoclast pool through the release of RANKL,^79^ particularly within the bone cortex.^80^ In contrast, when PTHR1 is deleted, RANKL release and subsequent osteoclastogenesis do not occur.^81^

Interestingly, in addition to BMU activation, three hours after PTH exposure, demineralization of the bone matrix surrounding osteocytes accompanied by compensatory mineral deposition has been observed.^82^ The same outcome was observed in cases of excess PTH concentrations both in rats^83^ and humans^84^ and under PTH modulation after exercise.^85^ This process is mediated by osteocytes and is called perilacunar remodeling (i.e., “osteocytic osteolysis”); it was initially recognized during lactation. Perilacunar remodeling has been associated with the activation of PTH-related peptide (PTHrP)–PTHR1 signaling, which induces matrix resorptive activity in osteocytes similar to that in osteoclasts.^86^ Perilacunar remodeling integrated with bone remodeling contributes to the maintenance of bone quality. Perilacunar remodeling is an additional component of the long-term error correction mechanism to maintain plasma calcium levels, in addition to the homeostatic regulatory mechanism in osteoclasts (RANKL-mediated). The efficiency of this additional and independent component of bone remodeling in calcium homeostasis under PTH regulation remains unclear because of the lack of a relationship between serum PTH and osteocyte lacuna characteristics.^87^

Nevertheless, as has been recently discussed,^88^ osteocytes *per sé* function independently of associated endocrine loops and exhibit an evolutionary advantage in mineral homeostasis, which is particularly highlighted in the rapid minute-to-minute regulation of the BEF-[Ca^2+^] in teleosts,^89^ as well as in mammals.^26,90^

Osteocytes, through PTHR1 activation, might counteract age-related bone mass loss. In fact, during aging, as well as under the action of several stressors [see Farr et al.^91^ for a review], senescent osteocytes accumulate in the bone microenvironment and acquire a distinctive proinflammatory secretome, termed the senescence-associated secretory phenotype (SASP),^92^ which leads to imbalanced bone remodeling with increased resorption and decreased formation. When PTHR1 was deleted in mouse osteocytes in vivo (Dmp1-PPR^KO^), the affected mice displayed age-dependent osteopenia related to a decrease in osteoblast activity with a parallel rise in osteoclast number and activity. The imbalanced bone remodeling in these animals was partially due to a sclerostin-dependent decrease in osteoblast activity and the lack of osteocyte protection from oxidative stress.^93^

### Estrogen

Osteocytes respond to estrogen by producing the protein semaphorin 3A (Sema3A). Sema3A released into the bone microenvironment binds to its receptor on osteocytes and promotes their survival. The autocrine loop initiated via Sema3A is mainly triggered in the mature cell stage, which is the dominant characteristic of cells in the adult skeleton and is critical in bone remodeling balance, counteracting age-related bone loss. Autocrine loop impairment caused by estrogen deficiency induces an osteoporosis phenotype.^94^ In addition to its effects on bone remodeling, estrogen regulates mechanotransduction in osteocytes by affecting FFSS,^95^ Wnt/β-catenin expression,^96^ and Cx43 expression.^97^ Its removal triggers osteocyte apoptosis and alters the oxidative microenvironment, leading to the loss of osteocyte resistance to oxidative stress.

### TGF-β1

Recently, osteocyte-intrinsic TGF-β1 signaling was discovered as a regulator of lacuno-canalicular remodeling. The suppression of TGF-β1 signaling in osteocytes, either pharmacologically (by TGF-β receptor type I kinase inhibitors) or genetically (by specific deletion of the receptor TβRII in DMP1-cre mice), leads to the reduction osteocyte dendrites length and total lacuno-canalicular area. The deterioration of the lacuno-canalicular network was accompanied by the decrease in the gene expression of Sost, and metalloproteases Mmp2, 13, 14, which are involved in the lacuno-canalicular remodeling, and by the reduction of fracture resistance in cortical bone despite no differences in cortical thickness and geometry, and increase in trabecular bone mass, likely due to the observed decrease in osteoclasts number and surface.^98^ The observation that lacuno-canalicular remodeling is an essential component of bone mechanical competence sustains the view that osteocytes play an evolutionarily conserved role in bone quality control.^98^

### 1,25(OH)_2_D_3_

In addition to its systemic effects, 1,25(OH)_2_D_3_ binds to VDR in osteocytes in an autocrine manner, and as they mature, osteocytes acquire the capacity to convert physiological levels of 25(OH)D to 1,25(OH)_2_D_3_.^99^ The exact role of 1,25(OH)_2_D_3_ in osteocyte metabolism is still uncharacterized. However, recent observations have pointed out that 1,25(OH)_2_D_3_ a) stimulates the production of fibroblast factor 23 (FGF23) in osteocyte-like cells;^100^ the hormone FGF23 is primarily involved in phosphate homeostasis and vitamin D synthesis; b) modulates matrix mineralization by downregulating dentin matrix protein-1 (DMP-1);^101^ and c) regulates osteocyte perilacunar remodeling and canalicular organization through the activation of matrix resorption genes.^102^

### Phosphate

The establishment of X-linked hypophosphatemia (XLH) mouse models, characterized by elevated serum FGF23 levels, which caused decreased 1,25(OH)_2_D_3_ levels and hypophosphatemia, led to the discovery of the role played by phosphate in osteocyte functioning, which is triggered in response to disruption of plasma phosphate homeostasis.^103^ How osteocytes sense phosphate levels and subsequently regulate perilacunar remodeling^103^ and FGF23 and 1,25(OH)_2_D_3_ synthesis^104^ is still unclear. However, as has been previously discussed,^105^ the phosphate-sensing mechanism in osteocytes may involve the activation of FGFR1 and high-affinity Na^+^-Pi cotransporters.

### Calcitonin

Osteocytes express specific calcitonin receptors, which are progressively lost with age.^106^ Calcitonin can potentially modify the osteocyte production of sclerostin and FGF23.^107^

### Irisin

Irisin, a myokine produced by muscles, reduces osteocyte apoptosis and increases osteocyte number.^108^ These effects suggest a regulatory loop involving osteocytes and muscle metabolism, particularly since osteocytes inhibit skeletal muscle differentiation by producing a large number of cytokines, which might permeate the periosteum or diffuse into the circulation to negatively regulate myogenesis^109^.

These findings indicate that osteocytes can be considered the “social” coordinators of bone cells because they are responsible for maintaining bone physiological responsiveness to mechanical and metabolic demands. When osteocytic coordination fails, severe osteoporosis develops.^110^

## Bone lining cells (blcs), canopy cells and pericytes: composition of the vascular bone remodeling compartment (brc)

BLCs, derived from the final differentiation of osteoblasts, are located on quiescent trabecular, endosteal, and endocortical surfaces at the end of bone formation. BLCs are a cell population that differs from both marrow cells^111^ and osteoblasts. BLCs are flattened and exibits lower synthetic activity, with little cytoplasm or endoplasmic reticulum,^112^ but they retain a social attitude. Similar to osteocytes, BLCs express intercellular adhesion molecule-1 (ICAM-1) to maintain functional contact with osteoblasts, osteoclast precursors^113^ and mature osteoclasts.^114^ They cover quiescent bone surfaces that are not undergoing remodeling, but they are not “quiescent” as the older literature suggests. Although their specific function has not yet been fully defined, BLCs might form an epithelial-like membrane that functions as an ion partition system between bone and systemic extracellular fluids in Ca^2+^ homeostasis.^26,90,115^ Indeed, the BLC membrane expresses tight junction membrane proteins that are responsive to metabolic demands such as chronic metabolic acidosis.^116^ Moreover, BLCs stain positive for osteoblast markers such as alkaline phosphatase, (ALP) osteocalcin (OCN), and osteonectin, in agreement with their osteogenic potential. BLCs are covered by a thin layer of mesenchyme-derived cells called bone marrow envelope (BME) cells that morphologically resemble BLCs and, similar to BLCs, are considered osteoprogenitors.^2^

**Fig. 3:**
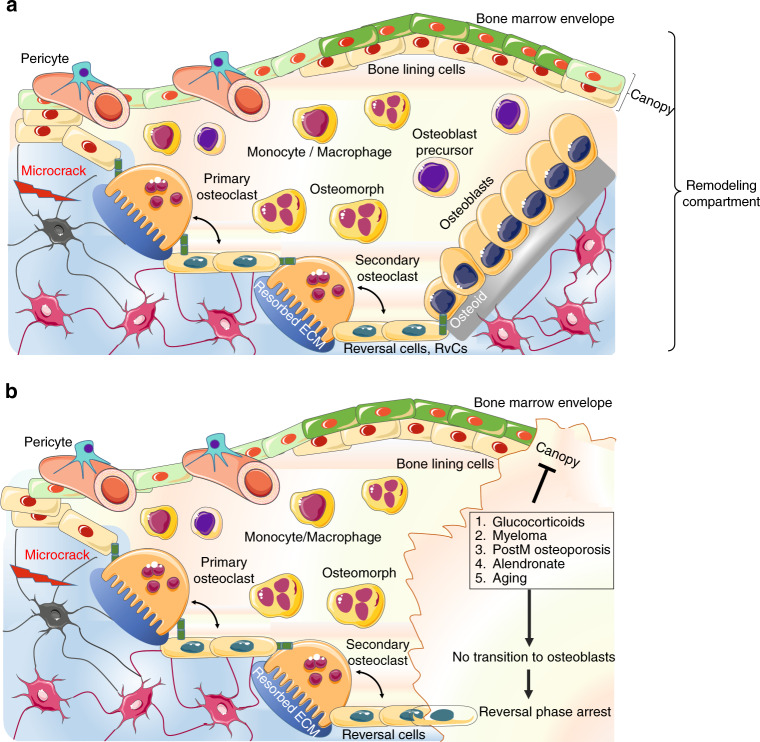
The bone remodeling compartment (BRC). **a** The BRC provides the correct microenvironment to link bone formation and resorption through local signaling. The bone marrow envelope (BME) is a layer of cells of mesenchymal origin and a reservoir for osteoprogenitors that covers the layer of bone lining cells (BLCs). Once remodeling is initiated, osteoclasts lift the BME from the BLC, inducing BME cells to form a structure called the canopy. The canopy separates the remodeling site from the remainder of the bone marrow to allow osteoclast and osteoblast precursors to enter the blood compartment. After resorption, osteoclasts either undergo apoptosis or dedifferentiate into osteomorphs. The resorbed surface is then colonized by secondary osteoclasts and reversal cells. Reversal cells are osteoblast progenitors that digest fibrillar collagen remnants, similar to BLCs. Secondary osteoclasts and reversal cells provide the basis for the recruitment and expansion of osteoblastic pools (**b**) except under certain counteracting conditions, such as glucocorticoid or alendronate treatment, myeloma, or postmenopausal osteoporosis

When the remodeling starts, BME cells and BLCs form a protective structure known as the canopy, which separates osteoclasts and osteoblasts from bone marrow^117^ (Fig. 3). At the bone remodeling site, BLCs disconnect from the underlying osteocytes through gap junction disruption, and digest fibrillary collagen, the most abundant component in the bone extracellular matrix (ECM), which is tightly packed in a helical structure to provide mechanical stability and confer resistance against proteolysis.^118^ To be degraded, fibrillary collagen usually requires the activation of the cysteine protease cathepsin K and members of the matrix metalloproteinase (MMP) family.^119^ While osteoclasts express both of these enzymes types, which contribute differently to bone resorption,^120^ BLCs do not express cathepsin K but can efficiently remove nonmineralized fibrillary collagen (present either on the quiescent bone surface or in resorption lacunae after osteoclastic activity)^113^ mediated through their highly active MMPs.^121^

The generation of the canopy allows the formation of the bone remodeling compartment (BRC), which is considered an essential component of the BMU itself, as it provides a microenvironment to link bone formation and resorption through local signaling.^122^

The BRC is generated in the proximity of the microvasculature^123^ (Fig. 3). Although it is unclear how the BRC is formed, several lines of evidence have suggested that the BRC favors a direct connection between bone cells and sinusoids, which might provide reservoirs of osteoprogenitors.^122^

In support of this hypothesis, an increased presence of capillaries covering human cancellous bone has been found at remodeling sites in an area within 50 µm from the canopy surface.^124^ This coverage has also been observed on cortical bone remodeling sites.^4^

The tight link between BRCs and capillaries, which are coated with pericytes, allows capillary-BRC canopy interactions to induce BMU development at bone remodeling sites^117^ (Fig. 3). Pericytes, first identified as cells covering microvessels/capillaries and maintaining local homeostasis and angiogenesis, support the mesenchymal niche and can be considered osteoprogenitors.^125^ It has been hypothesized that pericytes first move from capillaries to the canopy and then to the bone surface, where they differentiate into mature osteoblasts during bone remodeling*.*^126^ It is also plausible that the vascularization of the BRC favors the recruitment of osteoprogenitors circulating in the peripheral blood*.*^127^

Recently, a specific capillary subtype that supports perivascular osteoprogenitor differentiation by producing Nogging via Notch/Dll 4 signaling has been termed type H vessel, which express both CD31 and Endomucin at high levels. Type H vessels are surrounded by abundant osteoprogenitors (Osterix-positive cells) and are located both in the metaphysis, close to the growth plate, and in the diaphysis (periosteum and endosteum).^128,129^ During bone remodeling, the release of platelet-derived growth factor type BB (PDGF-BB) from preosteoclasts induces an increase in endothelial cell recruitment and assembly, with a subsequent rise in the number of type H vessels. Simultaneously, PDGF-BB triggers the recruitment of osteoprogenitors from vessels to the bone surface.^130^ The formation of H vessels is supported by the production of the slit homolog 3 (SLIT3) protein in osteoclasts,^131^ which also enhances H vessel branching.^131–133^

Type H vessels respond to the administration of intermittent anabolic PTH (iPTH), which promotes the detachment of leptin receptor-expressing (LepR^+^) cells, which are pericytes that can differentiate into osteoprogenitors,^134^ from CD31^hi^/Edm^hi^ vessels. Pericyte migration contributes to the osteoprogenitor recruitment to the BRC during PTH-induced bone remodeling.^135^ Notably, anabolic responses to iPTH as well as sclerostin inhibitor treatments, decline with time,^136^ as it is conceivable that the precursor pool tends to be exhausted after major stimulation.

BLCs do not exclusively represent a final differentiation stage of the osteogenic line; they can also be osteoblast precursors.^137^ A study by Matic et al.^137^ led to the development of a model in which osteoclasts initiate osteoprogenitor cell expansion (see following section) by activating osteoprogenitor reservoirs located in proximity to the eroded bone surface consisting of BLCs, canopy cells and pericytes.^120^

Hence, BLCs favor the initiation of bone formation by supporting the recruitment of bone-forming osteoblasts from the canopy upon the release of osteoclastic factors supporting bone formation, independently of bone resorption (see following section). The osteoclast-canopy interface is therefore the physical site where the coupling of bone resorption to bone formation occurs.^117^

## Osteoclasts

### Resorbing osteoclasts: osteoclastogenesis and excavation of resorption lacunae

Osteoclasts are responsible for bone resorption, a process that is accomplished in a fairly short time relative to that required for bone formation. Osteoclasts originate from the hematopoietic monocyte-macrophage lineage, residing within the granulocyte-macrophage colony-forming unit. Osteoclastogenesis starts in the bone marrow upon the release of RANKL and macrophage colony-stimulating factor (M-CSF) from osteocytes and vascular endothelial cells close to the bone surface.^138^ It then extends toward the bone surface through the layer of BLCs along an “osteogenesis route”, likely guided by physical (i.e., collagen) and biochemical (i.e., sphingosine-1-phosphate, S1P) signals.^139^ In vitro experiments have shown that osteoclasts access bone surfaces by directly displacing the cells present on the bone surface.^140^ TGF-β1 is important for inducing cytoskeletal reorganization via the p38 pathway in osteoblastic cells, which elongate to generate cell-free areas.^141^ The inhibition of c-src and subsequent actin organization in osteoclast precursors prevent osteoclasts from migrating through the osteoblast layer.^142^ Metalloproteinase (MMP) inhibitors prevent both osteoblast elongation and the subsequent generation of cell-free areas^143^ and directly act on osteoclasts by preventing their migration through the cell layer.^141^ However, in osteoclasts, MMP inhibitors did not affect actin organization *per sé*,^142^ although they had been previously demonstrated to prolong the podosome lifespan in these cells.^144^ MMP inhibitor administration fully prevented osteoclast recruitment in the diaphysis core in vivo^145^ as well as osteoclast migration through collagen in vitro.^146^ Among MMPs, MMP-14 and MMP-9 seem to play a major role in osteoclast migration. MMP-14, also known as MT1-MMP, is expressed in all skeletal cells but is more abundant in osteoclasts.^147^ MT1-MMP-knockout (KO) mice usually die couple of months after birth, and display a severe phenotype that includes delayed ossification, unclosed sutures and unremoved cartilage.^148^ MMP-14 is highly expressed on osteoclast podosomes,^149^ and it digests interstitial collagen as well as other ECM molecules.^150^ MMP-9-KO mice showed reduced osteoclast invasion into the core of developing bones,^151^ and in vitro, osteoclasts lacking MMP9 did not migrate.^152^ However, very recently, it has been demonstrated that osteoclasts lacking either MMP9 or MMP14 alone (in this case conditionally deleted in the myeloid population) did not show altered fusion and function, or any defects in bone resorption. Only the deletion of both MMP9 and MMP14 generated a reduction in bone resorption areas and in type I collagenolysis activity.^153^

The fusion of the earliest osteoclast precursors into multinucleated, mature osteoclasts is a critical process in osteoclastogenesis, as the number of nuclei per cell determines osteoclast aggressiveness.^154^ The presence of distinct giant hypernucleated osteoclasts *per sé* in patients undergoing long-term treatment with nitrogen bisphosphonates (NBPs), as discovered by Weinstein et al.^155^ and confirmed by others,^156^ did not necessarily indicate an increase in osteoclast aggressiveness. These osteoclasts indeed showed decreased resorption competency and prolonged apoptosis characteristics. A recent study with transmission electron microscopy demonstrated that NBPs first induce osteoclast apoptosis and then the formation of newly differentiated osteoclasts that can anchor the bone surface but cannot form a clear zone or ruffled border.^157^ NPBs induce osteoclasts to acquire a nonresorbing phenotype by inhibiting c-Src, which is essential for cytoskeletal construction,^158^ and by stimulating dendritic cell-specific transmembrane protein (DC-STAMP),^159^ which is necessary for cell fusion.

Under physiological conditions, osteoclast fusion requires immobility and heterogeneity between fusion partners; fusion can only occur at the bone surface and must be completed for successful resorption.^139^ As reviewed elsewhere,^160^ heterogeneity is an integral feature of osteoclast fusion and may be related to the capacity of fusion precursors to adapt to specific bone microenvironments. Fusion also requires RANKL, which is highly expressed by BLCs;^161^ osteoblasts in the growing skeleton; and osteocyte arborizations that reach the bone surface in adults.^51,52^ sRANKL or mRANKL produced by osteogenic cells binds to RANK on osteoclast precursors to activate intracellular signaling of tumor necrosis factor receptor-associated factors (TRAFs), especially TRAF 2, 5, 6, which are adapter molecules that trigger NF-κB activation.^162^ NF-κBs are transcription factors that were originally discovered as regulators of B lymphocyte differentiation^163^ and that play important roles in innate and adaptative immune responses.^164^ Mice lacking both NF-κB 1 and 2 display, among other features, severe osteopetrosis^165^ due to an accumulation of osteoclast precursors.^166^ NF-κBs can activate canonical signaling and noncanonical signaling pathways in osteoclasts. Canonical NF-κB signaling activation is very fast, initiated within one hour of RANKL binding, and leads to the induction of NFATc1 expression.^167^ NFATc1 is a transcription factor that plays a master role in osteoclastogenesis^168^ by regulating the expression of osteoclast-specific genes such as cathepsin K, DC-STAMP, and tartrate-resistant acid phosphatase (TRAP) or that of osteoclast-associated receptor (OSCAR) and other genes involved in OC resorptive functions.^169^ The role of the Nf-κB noncanonical pathway in osteoclastogenesis is controversial. Global deletion of single molecules involved in the Nf-κB noncanonical pathway did not affect osteoclast numbers in vivo,^170^ whereas conditional deletion of TRAF3, an inhibitor of the noncanonical pathway, in osteoclast precursors resulted in increased osteoclast formation and bone resorption in mice*.*^171^

The discovery of a forward signaling induced by RANKL binding to its receptor RANK on osteoclast precursors led to the development of denosumab (DMAB), a human monoclonal antibody that binds RANKL and inhibits osteoclastogenesis. Recently, a second receptor for RANKL, the leucine-rich repeat-containing G protein-coupled receptor LGR4, was discovered on the osteoclast membrane. The extracellular domain of LGR4 binds RANKL and inhibits NFATC1 expression, thus blocking osteoclastogenesis.^172^ Very recently, a variant of RANKL with changes in a few amino acids in the RANK binding site was developed. This variant still activates LGR4 signaling and inhibits osteoclastogenesis. Moreover, it has been observed that this variant acts as an immunogen triggering the production of anti‐RANKL antibodies, and its use might reduce the risks linked to the abrupt suspension of denosumab use.^173,70^ The balance achieved among RANK, RANKL, OPG, and LGR4 fine tunes bone resorption in the bone remodeling process, and the pharmacological modulation of this balance has critical therapeutic potential in osteoporosis.^172^

In addition to RANKL/RANK, LGR4 and M-CSF signaling^174^ guiding and balancing the early steps of osteoclastogenesis, other factors act in concert to determine the osteoclastic mature cell phenotype, including β3-integrin,^175^ NR4A1,^176^ immunoreceptor tyrosine-based activation motif-containing proteins (ITAMs), DNAX-activating protein and the Fcγ receptor.^177^ Additionally, Toll-like receptors (TLRs) expressed on osteoclast progenitors^178^ promote differentiation toward the mature cell phenotype, as observed in inflammatory osteolytic diseases.^179^

WNT proteins such as Wnt5A in the noncanonical pathway have been outlined as key elements in osteoclastogenesis. WNT5a produced by osteoblasts supports osteoclastogenesis by stimulating RANK expression in osteoclast precursors,^180^ promoting osteoclast fusion,^181^ and supporting actin ring formation and bone resorption *via* c-Src.^182^ Since WNT5a enhances osteogenic pathway of Wnt/β-catenin activation in osteoblasts,^183^ it was thought that WNT5a produced by osteoblasts might affect both resorption and formation. However, recent studies have led to modifications of this previous assumption as they demonstrated that, after RANKL stimulation, osteoclast precursors also produce and secrete a unique phosphorylated form of WNT5A. Furthermore, deletion of this phosphorylated form of WNT5a in mature osteoclasts led to reduced bone formation in male mice.^184^ Hence, it is speculated that in the BRC, osteoblast WNT5a first supports osteoclastogenesis; then, as the osteoclast precursors differentiate, WNT5a expression increases, leading to the subsequent enhancement of osteoclast fusion and activity. In later stages, WNT5a production by mature osteoclasts fosters osteoblastogenesis because WNT5a cooperates with canonical Wnt signaling. The subsequent expansion of the osteoblast pool might therefore fulfill the resorption cavity at the proper time and space, suggesting that Wnt5a is a clastokine that maintains osteoclast–osteoblast coupling in physiological bone remodeling.

The cytoskeletal and membrane organization of osteoclasts characterizes their differentiation state. Mature osteoclasts are polarized cells with an apical membrane facing bone and an opposite basolateral membrane facing plasma. Both apical and basolateral membrane domains are essential structures for resorbing osteoclasts.^185^ Critical functional features of the apical membrane domain are the sealing zone and the ruffled border. The sealing zone establishes contact with the bone surface and delimits the resorption lacunae (i.e., Howship’s lacunae). The ruffled border membrane allows large vesicle transport, the delivery of hydrochloric acid (by vacuolar-type H + ATPase^186^) and chloride ion channel-7 (CLC-7),^187^ and the release of several lysosomal proteases, such as cathepsin K, TRAP, and MMPs.^188^ After endocytosis, the products derived from matrix degradation are transported through the cell within vesicles for further intracellular degradation and exocytosed on the opposite site through the secretory domain at the basolateral membrane.^189^ Thus, the osteoclasts never lose their tight attachment to the bone surface when resorption occurs. The interaction with the matrix is mediated by the integrin complex α5β3, which recognizes RGD motifs in proteins such as fibronectin,^190^ osteopontin (OPN)^191^ and bone sialoprotein.^192^ By binding to RGD motifs, the α5β3 complex is activated and clusters at the ruffled border,^193^ thus allowing the activation of focal adhesion complexes and the formation of the sealing zone during resorption.^194^ It has been hypothesized that integrin β3 contains two motifs with different affinities (high and low) for Ca^2+^ concentrations. A normal Ca^2+^ concentration in BEF maintains low-affinity domain activation, which does not maintain a strong attachment between β3 and RGD. When Ca^2+^ concentrations diminish, such as when osteocytes are injured, the high affinity site is the only one activated; this motif induces αvβ3 clustering, which allows osteoclasts to strongly adhere to the matrix and initiate bone resorption.^195^

The overall fate of osteoclasts (i.e., recruitment, differentiation, fusion and apoptosis, see below), is tightly controlled by the process coordinating bone remodeling, in which osteoclasts are also providers of multiple coupling signals^196^ for osteoblast formation.

### Resorbing osteoclasts: the release of bone matrix factors

The bone ECM resorbed by osteoclasts contains inorganic and organic compounds. A growing body of evidence in ECM biology suggests that several organic proteins of the matrix regulate the quantum of BMU activity. While the only inorganic components of the ECM are apatite and trace elements, proteomic analysis have led to the identification of more than 100 organic ECM proteins,^197^ with collagen type I (Coll-1) and noncollagenous proteins (NCPs) being the major constituents. NCPs can bind growth factors, membrane receptors, and adhesion molecules, forming an intrinsic biochemical signaling network within each BMU. NCPs comprise several Gla proteins, including bone-Gla protein (i.e., Osteocalcin, OCN), matrix-Gla protein (MGP), protein-S, Gla-rich protein (GRP), periostin, and periostin-like factor (PLF), as reviewed elsewhere.^198^ In addition to OCN, whose hormonal effects were previously established in animal models,^199^ Gla proteins exhibit several functions supporting bone metabolism through γ-carboxylation-dependent and γ-carboxylation-independent mechanisms, ranging from the regulation of cell adhesion and activity to the modulation of calcium concentrations in the extracellular space. Gla proteins act in concert with glycoproteins such as osteonectin, thrombospondins and R-spondins, sialoprotein and matrix extracellular phosphoglycoprotein (MEPE) and DMP1 to control mineralization, synergistically with FGF23 and PHEX produced by osteocytes (see Lin et al.^200^ for review).

**Fig. 4:**
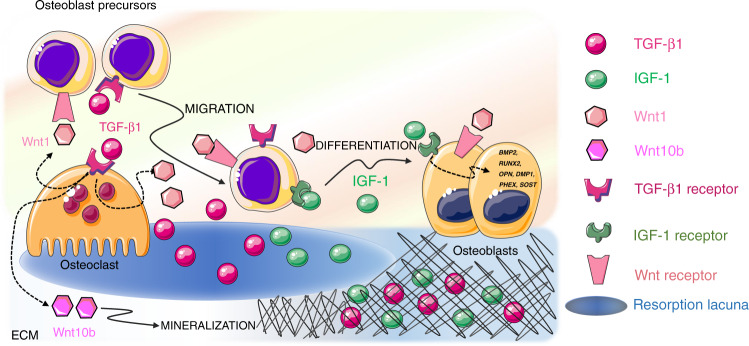
Released extracellular matrix (ECM) factors. Two major factors are released by the ECM upon bone resorption and cooperate to regulate bone remodeling and BMU activity: TGF-β1 and IGF-1. After release from the matrix, active TGF-β1 acts both on osteoblasts and osteoclasts to induce osteoblast precursor migration to the site of resorption and osteoclast production of Wnt1, which promotes osteoblast recruitment and/or differentiation at sites of bone resorption, and Wint10b, which promotes matrix mineralization. IGF-1 supports the recruitment of osteoblast progenitors and promotes osteoblast differentiation and matrix mineralization by inducing the transcription of osteogenesis‐related genes such as DMP1, PHEX, SOST, BMP2, RUNX2, OPN, and OCN

Two major factors released by the ECM influence bone remodeling and BMU activity: transforming growth factor-β (TGF-β) and insulin growth factor-1 (IGF-1). Together, TGF-β and IGF-1 influence the recruitment and differentiation of osteoblast lineage cells (Fig. 4), favor bone matrix mineralization, and regulate osteoclast activity. For these reasons, they are acknowledged as “coupling factors”.

#### TGF-β

TGF-β1 is one of the most abundant cytokines in the bone matrix.^201^ TGF-β1, which is a member of the TGF superfamily that includes bone morphogenetic proteins (BMPs), binds to specific TGF receptors (TGFR1-2) in osteoblasts, where it activates the synthesis of collagen and the expression of RUNX2, a master transcription factor regulating osteoblast proliferation and differentiation. TGF-β1 release and activation, resulting from the activity of metalloproteinases and interaction with integrins, are prerequisites for the exertion of TGF-β1 effects on bone cells. This occurs during bone resorption since osteoclasts lower the pH in resorption lacunae and secrete cathepsin K, thus releasing and activating TGF-β1.^202^ When osteoclasts actively resorb bone, TGF-β1 is released in the lacunae, thus playing a crucial role in the balance between bone formation and resorption. First, active TGF-β1 induces osteoblast precursor migration to the site of resorption^203^ without affecting the differentiation of cells.^204^ Since TGF-β1 alone is not able to promote osteoblast differentiation,^205^ cooperative pathways need to be activated to foster an osteogenic environment at resorption surfaces within the BRC. A cooperation pathway is provided by the osteoclast itself. In addition to activating and releasing TGF-β1 from the bone matrix, osteoclasts express specific receptors for TGF-β1. Osteoclastic TGF-β1 receptor signaling stimulates the production of Wnt1, which promotes osteoblast recruitment and/or differentiation at sites of bone resorption. Since impaired TGF-β1 receptor signaling in osteoclasts has detrimental effects on bone mass in mouse models, it is likely that the release of Wnt1 constitutes a coupling pathway.^206^ Moreover, TGF-β1 favors RANKL production in osteoblast precursors by inducing TRAF3 degradation. The expression of TRAF3, which is an inhibitor of noncanonical Nf-κB signaling, in osteoblast precursors favors the stabilization of β-catenin and, therefore, their differentiation into mature osteoblasts. In the absence of TRAF3 expression, Nf-κB noncanonical signaling is activated with an increase in RANKL production and therefore is associated with osteoclastogenesis.^207^ With matrix resorption progression, the concentration of TGF-β1 increases, and TGF-β1 activates a negative feedback loop in osteoclastogenesis. High TGF-β1 expression inhibits the migration of osteoclast precursors^208^ by suppressing RANKL expression and by stimulating OPG production in osteoblasts.^209^ A reduced RANKL/OPG ratio indeed results in reduced osteoclastogenesis. In the absence of osteoclast survival factors such as RANKL, TGF-β1 leads to osteoclast apoptosis via the upregulation of Bim expression.^210^ This proapoptotic effect of TGF-β1 on osteoclasts is consistent with the crucial role it plays in the reversal of bone resorption to bone formation. Indeed, TGF-β1 release enhances Wnt10b expression and secretion to stimulate osteoblast-directed mineralization. TGF-β1 therefore has a dual effect on osteoblasts by directly recruiting their progenitors to the bone surface and by indirectly promoting matrix mineralization through osteoclast-derived Wnt10b.^211^

#### IGF1

IGF-1 is the most abundant growth factor deposited in the bone ECM.^212^ Osteocytes in particular secrete large amounts of IGF1, which is incorporated in the bone matrix.^213^ Since IGF-1 is produced under loading, it might contribute to the mechanotransduction process.^214^ Whether released by cell secretion or bone ECM degradation, IGF1 is a local autocrine/paracrine regulator of bone remodeling and does not contribute to the circulating IGF-I pool. IGF-1 acts primarily as a migratory signal and as a differentiation factor for osteoblast precursors, rather than as a proliferation factor. IGF-1 recruits progenitor cells of the osteoblastic lineage^215^ and promotes osteoblast differentiation and matrix mineralization by upregulating the osteogenesis‐related genes DMP1, PHEX, SOST, BMP2, RUNX2, OPN, and OCN.^216^ Moreover, IGF-1 released from the bone matrix during bone resorption establishes an osteogenic microenvironment by activating mechanistic target of rapamycin (mTOR) signaling,^217^ which is critical for cellular energy metabolism and cell migration.^218^ Recent studies have confirmed that IGF-1-dependent activation of mTOR induces human dental pulp stem cell (DPSC) differentiation toward an osteoblastic phenotype.^219^

Since TGF-β1 and IGF1 do not remain accessible long enough to affect the complete refilling of the resorption cavity,^220^ their action might be limited to the early phases of remodeling, when osteoclasts are still actively resorbing the bone matrix.

## Anabolic osteoclasts: a bridge from bone resorption to formation

The resorption of the bone ECM is necessary, but not sufficient, to induce the stimulus provided by osteoclasts to osteoblasts during remodeling. Currently, available evidence indicates that osteoclasts can support bone formation independent of their resorption activity. The paradoxical concept of “anabolic osteoclasts” was articulated after the original observation that human osteoblasts exposed to conditioned media from human osteoclasts increased bone nodule formation.^221^ The finding that the transplantation of nonfunctional osteoclasts in irradiated skeletally mature 3-month-old wild-type mice induced a high trabecular bone volume, increased bone strength, and an increased bone formation rate in trabecular bone^222^ supports this concept. The anabolic role of osteoclasts can be observed in both humans and mice with osteoclast-rich osteopetrosis (OPT), a disease resulting from mutations of either the V-ATPase subunit or the ClC-7 chloride channel that abrogate the osteoclastic acidification process, which is essential for resorption activity. Under these conditions, no factors stored in bone were released from the unresorbed bone matrix, but the long-surviving osteoclasts, fused into large abnormal multinucleated cells, provided an osteogenic milieu.^223^ In fact, in models of osteoclast-rich OPT, bone formation is maintained or enhanced despite the impairment of resorption activity,^224^ thus providing evidence that nonresorbing osteoclasts promote/maintain bone formation. Findings following the pharmacologic blockage of cathepsin K in osteoclasts (Odanacatib) are in line with this view. In patients treated with Odanacatib, although osteoclast bone resorption was impaired, the osteoclast number was increased to be more than twofold higher than that after placebo treatment by month 60, and bone formation, after a transient reduction, was maintained.^225^

In contrast to “osteoclast-rich” OPT, active bone formation is lacking in “osteoclast-poor” OPT (due to TNFRSF11A mutations^226^) or dysosteosclerosis (DSS) (an OPT of unknown etiology^227^). Indeed, bone histomorphometric analysis showed no linear single or double tetracycline labels under these conditions.

Therefore, these “anabolic osteoclasts” might produce a pool of signals that activate bone formation and that can be a) released into the BRC, b) shuttled to osteoblasts, and c) transferred by receptor binding.

### Information released into the BRC

Several osteoclast-derived factors released into the BRC have been identified and validated in genetically modified mouse models. These factors include cardiotrophin-1 (CT-1),^228^ S1P,^229^ Wnt10B, BMP6,^230^ collagen triple helix repeat-containing 1 (CTHRC1),^231^ complement factor 3a (C3a),^232^ and leukemia inhibitory factor (LIF).^233^ However, not all of these identified factors are responsible for coupling in bone remodeling, nor are they produced only by osteoclasts due to macrophage contamination in vitro and the presence in vivo of immunocompetent cells within the remodeling compartment. Whether these factors exhibit similar effects in humans remains unclear; however, a recent study shed some light on this controversy. Bone biopsy samples taken from postmenopausal women treated with DMAB were analyzed by RNA sequencing, and the results were compared to those obtained from bone biopsy samples of untreated subjects. The comparison revealed potential osteoclast-derived coupling factors in humans: LIF, CREG2, CST3, CCBE1, and dipeptidyl peptidase-4 (DPP4), a highly conserved cell surface peptidase. The reduction in osteoclast-derived DPP4 in the DMAB-treated group was associated with a significant increase in glucagon-like peptide-1 (GLP)−1 in the serum compared to that in the placebo-treated group, suggesting that DPP4, in addition to its function as a coupling factor, might constitute a potential link between bone remodeling and energy metabolism.^234^

Mature human osteoclasts also secrete SLIT3, a chemorepellent originally identified as a regulator of axon crossing in the brain. Osteoclast-derived SLIT3 stimulates the migration and proliferation of osteoblast lineage cells via the activation of β-catenin and suppresses osteoclastogenesis in an autocrine manner.^131^ SLIT3 contributes to the initial establishment of the osteogenic environment after resorption and meets the requirements to be considered a coupling factor, as i) SLIT3 production increases during osteoclast differentiation and ii) SLIT3 is necessary for osteoblast precursor migration.^235^ Considering that bone greatly contributes to SLIT3 levels in plasma, SLIT3 might be considered a novel biomarker of bone turnover and a candidate for the treatment of osteoporosis because it plays active roles in both bone resorption and formation through its opposite effects.

### Information shuttled to osteoblasts

The coupling of osteoclasts to osteoblasts and related functions involve not only the secretion of factors in the BRC but also the production of specific EVs, released by bone cells. EVs have emerged as important intercellular regulators since they function as messengers from osteoclasts to osteoblasts, and vice versa.^236^

EVs exhibit an evolutionary advantage over the paracrine pathway activated by released factors because they can protect their message (mRNAs and proteins, including cytokines, etc.) from degradation and might therefore play critical roles in fine-tuning bone remodeling.

**Fig. 5:**
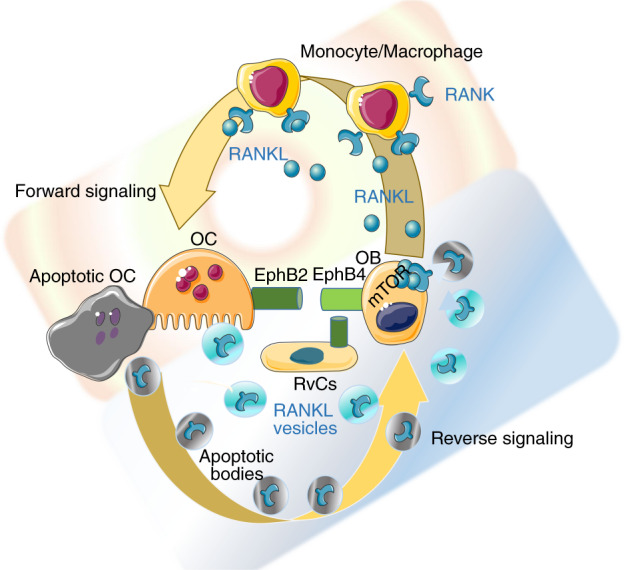
Anabolic osteoclasts. Osteoblasts stimulate osteoclastogenesis by producing RANKL as a forward signal. However, osteoclasts can act as anabolic cells by generating positive reverse signaling in osteoblasts. Both resorbing and apoptotic osteoclasts can release extracellular vesicles (EVs) that contain RANK. Once discharged from EVs, RANK binds RANKL clusters on the osteoblast membrane and activates osteogenesis via the mTOR pathway. In addition, EphrinB2 signaling from osteoclasts to EphB4 on osteoblasts/bone lining cells (BLCs)/reversal cells favors osteogenic differentiation

Osteogenic cells trigger hematopoietic precursor differentiation into osteoclasts by secreting RANKL. In addition, through a less-characterized mechanism, osteoclasts stimulate osteoblasts using the same RANKL–RANK pathway acting in reverse mode; that is, RANKL acts as the receptor. In fact, osteoclasts release EVs (between 25 and 120 nm in diameter) that carry RANK. These vesicles were initially thought to be negative paracrine regulators of osteoclastogenesis.^237^ However, a recent study demonstrated that vesicular RANK binds to RANKL on the osteoblast surface (and possibly on osteocytes) and triggers the mTOR-dependent stimulation of Runx2^238^ (Fig. 5). This finding suggests a previously unknown scenario in which actively resorbing osteoclasts promote the differentiation of neighboring osteoblast precursors into mature bone-forming cells. The physiological importance of the messages shuttled by EVs has been confirmed by the observation that osteoblasts do not efficiently deposit bone ECM when RANKL reverse signaling is suppressed.^238^

The discovery of the reverse signaling induced by RANK on osteoclasts binding to RANKL on osteoblasts indicates new pharmacological possibilities. Interestingly, the administration of a specific peptide, W9, to prevent RANKL-RANK signaling has been shown to exert a bone anabolic effect in vivo by inducing uncoupling.^239^ Therefore, DMAB may activate RANKL reverse signaling,^240^ explaining the continuous increase in bone mineral density (BMD) observed at the 10-year follow-up of DMAB-treated individuals.^241^ However, experimental evidence supporting the hypothesis of the bimodal effects of DMAB on RANKL-RANK signaling during bone remodeling is lacking because remodeling-based bone formation is severely suppressed in the femur of DMAB-treated subjects.^242^ It is more likely that DMAB blocks RANKL expressed on bone marrow mesenchymal stem cells (BMSCs),^243^ through which it negatively regulates osteogenic differentiation.^244^ According to a hypothesis suggested by Wang et al.,^243^ the DMAB-related abrogation of RANKL-induced inhibition of osteoblast precursors might underlie the activation of modeling-based bone formation^242^ and sustain the increase in BMD over time, as previously observed.^241^

RANKL monomers expressed on osteoblasts are not activated by RANK^238^ or by OPG, as indicated by the deletion of RANK or OPG failing to reduce bone formation.^245^ In contrast, the multimeric assembly of RANKL can activate reverse signaling. In fact, OP3-4, a RANKL-binding peptide that induces the clustering of RANKL on the cell membrane, induces the activation of RANKL reverse signaling.^246^ RANKL clustering on early osteoblasts may therefore be required for the activation of RANKL reverse signaling in resorption lacunae, similar to the effect of shuttling RANK in osteoclast EVs during bone remodeling (Fig. 5) or that of an anti-RANKL antibody-containing leucine zippers, which induce trimer formation.^238^

Some of the exosomes released by osteoclasts at the end of the two-week cell life span contribute to signal transfer during remodeling. In fact, apoptotic osteoclasts produce large amounts of apoptotic bodies. Apoptotic bodies are subcellular membrane-bound EVs containing fragments of nucleus, endoplasmic reticulum and mitochondria that assemble in a random manner and are involved in intercellular crosstalk.^247^ Osteoclast-derived apoptotic bodies contain osteoclast differentiation factors, such as RANK and RANKL (Fig. 5). In contrast, osteoblast-derived apoptotic bodies contain specific osteogenic factors, such as BMPs, OPN, OCN and bone ALP (BALP). By mapping the whole-proteome signatures of osteoclast-derived apoptotic bodies, the apoptotic bodies content was confirmed to be consistent with that of their parent cells in terms of both proteome signatures and biological functions.^248^ During remodeling, osteoclast-derived apoptotic bodies containing miR-214-3p are delivered to osteoblasts and serve as intercellular messengers that inhibit osteoblast differentiation and subsequent bone formation.^249^ Recently, the roles of osteoclast-derived apoptotic bodies have been more precisely characterized, and their effects have been found to be determined by the differentiation state of parental cells.^250^ For example, apoptotic bodies derived from mononuclear preosteoclasts undergoing apoptosis deliver PDGF-BB to recipient endothelial progenitor cells (EPCs), and since PDGF-BB is a proangiogenic factor,^130^ preosteoclast derived apoptotic bodies promote angiogenesis. However, apoptotic bodies derived from multinucleated mature osteoclasts promote osteogenesis via RANKL reverse signaling.

Therefore, in addition to well-studied osteoclast–osteoblast coupling, the involvement of osteoclast-derived apoptotic bodies promotes the transition from bone resorption to formation during bone remodeling. Although the mechanism by which apoptotic bodies delivery is controlled within each BMU is unclear, mature osteoclasts derived apoptotic bodies further connect the disappearance of osteoclasts from resorption lacunae with the incoming osteogenic process involving osteoblast recruitment, migration and differentiation, contributing to the reversal of cellular activity in reverting from bone resorption to bone formation.

### Information transferred by receptor binding

The communication route between osteoclasts and osteoblasts involves a bidirectional signaling pathway mediated by EphrinB2‐EphB4 that links the suppression of osteoclastogenesis to osteogenesis within each BMU.^251^ EphrinBs are transmembrane proteins with cytoplasmic domains that are preferential ligands for the tyrosine kinase receptor EphB. EphrinB2 expression in osteoclasts is associated with RANKL-induced osteoclast differentiation, whereas EphB4 is constitutively expressed in osteoblasts. Signaling from EphrinB2 in mature osteoclasts to EphB4 in osteoblast precursors stimulates osteogenic differentiation (Fig. 5), whereas signaling from EphB4 in osteoblasts to EphB2 in osteoclasts inhibits the differentiation of osteoclast precursors. EphrinB2–EphB4 bidirectional signaling must function locally and requires direct cell‐cell contact since both receptors are anchored to the cell membrane, as shown with goldfish scale model of bone remodeling.^252^ In humans, the role of EphrinB2 and EphB4 in bone remodeling has been debated because osteoclasts actively resorbing bone, and osteoblasts forming bone do not directly get in contact in the BRC. As a result the cell–cell interaction mediated by EphrinB2 and EphB4 might be limited to the precursor stage, when osteoclastogenesis and osteoblastogenesis are simultaneously regulated, or (as suggested previously^253^) to the mature osteoclast stage, in which direct contact is realized with the BLCs surrounding the canopy. Under PTH1R activation, EphrinB2 expressed on osteoclasts has been suggested to affect EphrinB4 on the osteoblast/BLC surface to induce osteoblast commitment and promote osteoblast differentiation.^254^

## Intermediate cell phenotypes: osteomorphs and reversal cells tuning and coupling bone remodeling

The orderly genesis and apoptosis of both osteoclasts and osteoblasts are essential for physiological bone homeostasis during bone remodeling. Possible intermediate phenotypes may be appropriate targets for coupling factors by providing a temporal connection between osteoclasts and osteoblasts. In the traditional view, osteoclasts were thought to live for two weeks^11^ before undergoing apoptosis; it was therefore assumed that during bone remodeling, the osteoclast resorption phase within a single BMU was temporally limited by the osteoclast life span, ending with the completion of its bone-resorbing activity. However, the observation that osteoclasts can survive longer by fusing with circulating monocytes with access to BRC^255^ has challenged this view. While osteoclast apoptosis is a rare event requiring high energy expenditure for the removal of the apoptotic debris, osteoclast disassembly into smaller unresorbing cells, which can revert to functional osteoclasts when needed, is a more efficient process. Two observations support the latter view: first, primary osteoclasts have been identified at the cutting cone and secondary osteoclasts have been shown to establish a functional link with reversal cells (Figs. 1 and 3) as focal bone remodeling progresses within a single BRC,^256^ and second, osteoclasts have recently been shown to be recycled into osteomorphs. Osteomorphs have been described as highly motile osteoclast daughter cells that remain in the adjacent bone marrow and retain the ability to fuse back into functional osteoclasts.^257^

The discovery of a transient cell stage of osteoclasts (i.e., the osteomorph) that can undergo phenotype reversion, analogous to what occurs in osteoblasts (i.e., BLCs), implies the presence of two reacting components of the BMU that can rapidly adjust the operational process of bone remodeling. This implication is clinically relevant to osteoporosis therapy. For example, BLCs can react to PTH exposure by quickly reverting to osteoblasts and thus expand the osteoblastic pool^258^ to fill resorption lacunae, similar to process in the “anabolic window” after teriparatide and abaloparatide administration.^259^ Similarly, exposure to RANKL might allow osteomorphs to rapidly fuse and recycle back into functional osteoclasts. Since RANKL inhibition by DMAB results in osteomorph accumulation,^257^ the reconstitution of RANKL signaling, as occurs upon DMAB discontinuation, can activate massive bone resorption over a very short time scale, leading to rebound fractures.^260^ This rebound effect is not fully inhibited by bisphosphonate therapy,^261^ probably because osteomorphs first accumulate at bone sites and are thus not accessible to potent bisphosphonate treatment. After osteoclasts are no longer active and disappear, the Howship’s lacunae remain covered with undigested nonmineralized collagen matrix. Mononucleated cells colonize these eroded surfaces (ESs), covering 80% them.^262^ These cells, called reversal cells,^263^ lack specific cell identification markers. The available literature has identified the cells appearing at the resorption lacunae as osteoclasts^264^ or, more recently, as osteoblasts.^265^ Indeed, the reversal cells express osteoblast lineage cell markers such as Runx2, ALP, and Col3, but not TRAcP or CatK.^265^ Indeed, reversal cells express osteoblast markers such as Runx2, ALP, and Col3 but not TRAcP or CatK.^265^ Abdelgawad et al.^266^ showed that the immunoreactivity of these cells for osteoclast-derived TRAcP is attributable to TRAcP taken up by osteoblast lines from neighboring osteoclasts and does not represent the acquisition of an osteoclastic phenotype. Moreover, Abdelgawad et al.^266^ observed that early reversal cells, located in close proximity to resorbing osteoclasts and forming direct cellular contacts with neighboring osteoclasts through short cytoplasmic extensions, are osteoblast progenitors with the capacity to digest fibrillar collagen remnants, similar to BLCs.^113^ In particular, reversal cells have been shown to be distributed among osteoclasts in the resorption surface far from the pockets of osteoclasts at the cutting cone tip. New resorption events might occur after the primary excavation of the resorption cavity/canal when reversal cells have colonized ES.^256^ In contrast, osteoclasts have never been observed at the bone formation wall, suggesting that bone resorption continues until bone formation takes place. Lassen et al.’s^256^ findings indicate that a pure reversal period, as commonly depicted following a halt in resorption,^8^ does not take place. In contrast, it is conceivable that the expanded pool of reversal cells, initially interacting with bystander osteoclasts in the same time and space, gradually switch off further resorption and form an osteogenic environment, activating bone formation later, when osteoclasts are completely absent.

The dynamic events described here support the view that the operational process of bone remodeling is a continuous physiological entity. All events, from the formation of the bone remodeling compartment with canopy cells to the colonization of a reversal zone by reversal cells in the early period of bone remodeling, generate the environment necessary for the recruitment and expansion of the osteoblast pool and subsequent refilling of resorption lacunae (Fig. 3).

## The osteoblastic cells pool: recruitment, expansion and osteocytogenesis

Osteoblasts are cuboidal-shaped cells on bone surfaces. Osteoblasts have traditionally been considered the cell responsible for the production of the organic components of the bone matrix and its consequent mineralization because they synthetize and secrete matrix components such as collagen type I, OCN, osteonectin, bone sialoprotein, OPN, proteoglycans, and ALP.^267^ In vivo, a set of osteoblasts, all with similar morphologies, lay down osteoid on active bone-forming surfaces*.*^268^ The dense endoplasmic reticulum of these cells indicates high synthetic activity, and the extensive contacts among these osteoblasts, osteocytes and BLCs indicate their extensive intercellular connections.^269^

Marotti et al.^270^ hypothesized that in human bone physiology, osteoblasts can participate in two different types of osteogenesis: static and dynamic osteogenesis. Static osteogenesis occurs in the mesenchymal tissue to initiate intramembranous ossification to generate primary trabecula during growth. This osteogenesis type is thus independent of the loading environment and will be subject to later osteoclast resorption. Static osteogenesis involves stationary osteoblasts, which are pluristratified and polarized in all directions. These osteoblasts differentiate into osteocytes in the exact same site where they had been originated from the mesenchymal precursors.^271^ Dynamic osteogenesis takes place to thicken the primitive trabeculae generated in the context of static osteogenesis, and it is therefore the type of osteogenesis activated in response to metabolic and mechanical demand. The osteoblasts participating in dynamic osteogenesis are arranged in an epithelial-like manner within a single cell layer. They all are polarized in the same direction, and they maintain contacts with osteocytic dendrites as they move from the mineralization front toward the vascular surface.^271^ In the adult skeleton, physiological bone remodeling involves only dynamic osteogenesis, which refill resorption lacunae.

Osteoblastic cells contribute not only to osteogenesis but also to osteoclastogenesis by producing RANKL. The same osteogenic cells may accomplish this dual function at different stages of maturation. Immature osteoblasts respond to bone regulatory factors in a pro-osteoclastogenic manner, but during the maturation process, they lose RANKL expression and do not support osteoclastogenesis.^272^ Moreover, as cells mature, they secrete more ECM and exert local inhibitory control over osteoclastogenesis by releasing the osteoclastogenic inhibitor OPG.^77^ Osteoblasts are considered the major sources of OPG in cancellous bone, acting in concert with osteocytes and other cells to modulate the RANKL/RANK ratio and reduce resorption by osteoclasts. This inhibitory activity is mandatory for the correct shift from bone resorption to bone formation and protects newly formed bone against untimely resorption.^77^ Cawley’s study stressed the concept “*that the local concentration of OPG near the cell surface is a key factor in its ability to block the activity of RANKL*”.

The osteoblast lineage derives from the differentiation of mesenchymal cells in the stromal compartment of bone marrow (BM). The bone marrow stroma contains self-renewing, multipotent progenitors that can give rise to osteoblasts, thus ensuring a reservoir of bone-forming cells for bone growth, modeling and remodeling.^273^ Anatomically, the BM stroma is part of the skeleton and is present in all bones except auditory ossicles, which therefore do not undergo remodeling.^274^ The definition of skeletal stem cells and their physiological significance and terminology associated with them have been debated.^273–275^ A self-renewing stromal progenitor, originally referred to as an “osteogenic” or “stromal”/”stem” cell (BMSC), corresponds to a specific type of perivascular cell in mammalian bone marrow. The characterization of pericytes as multipotent progenitors is evolving. Nevertheless, Sacchetti et al.^276^ pointed out that only BM vessel-residing cells defined as pericytes have the ability to differentiate into osteoblasts during physiological bone remodeling. Since tissue-specific mesodermal progenitors show tissue-specific commitments in vivo, the native skeletogenic potential is thus intrinsic to the progenitor/stem cell set found in the skeleton.^277^

Upon the initiation of bone remodeling, as previously discussed, the formation of BRCs by BLCs/canopy cells establishes a direct connection with the vascular sinus closely covering bone-forming surfaces, allowing pericyte detachment and differentiation toward the osteogenic phenotype.

Cells with true osteogenic potential can also circulate.^278^ Cells with multiple potential differentiation routes similar to those of BMSCs can enter the circulation, and although the mechanism by which they enter the circulation is unclear, they circulate in physiologically significant numbers, correlate with markers of bone formation and increase during bone growth.^127^ Characterization of these cells deserves further study, and circulating skeletal progenitors might form an additional pool of osteoblast precursors for bone remodeling and/or tissue repair. In fact, fractures result in the mobilization of mesenchymal osteogenic progenitors into the circulation to promote fracture healing, even in very old patients.^279^ Although circulating osteoprogenitor fate and function are determined by the homing of these cells to focal sites and their exposure to microenvironment signaling,^280^ their presence *per sé* in the blood has important implications for regenerative medicine.^281^

The average lifespan of active osteoblasts is 3 months.^4^ When bone matrix formation ends and the subsequent matrix mineralization process completes the primary phase, mature osteoblasts undergo three possible fates. Between 60% and 80% of osteoblasts at resorption lacunae die *via* apoptosis*.*^282^ Cells at all differentiation stages undergo apoptosis, which continues throughout the osteoblast life span and is regulated by Wnt signaling, PTH and mechanical stimulation.^61^ Osteoblast apoptosis is key to regulating the extent and duration of bone formation and is the target of catabolic and anabolic regulatory factors affecting bone mass. Apoptosis exerts a significant effect on the number of osteoblasts producing bone matrix at a bone formation site.^283^ The balance between the expression of proapoptotic factors Bim and Bak and the prosurvival factor Bcl determines the osteoblast life span and, therefore, bone volume and strength.^284^ Estrogens and androgens also exert effects on bone cell life span. In particular, they exert antiapoptotic effects on osteoblasts and osteocytes and proapoptotic effects on osteoclasts. Interestingly, estrogen receptors or the androgen receptor can transmit antiapoptotic signals with similar efficiency, regardless of whether the ligand is an estrogen or an androgen.^285^ p53, a potent tumor suppressor, is also critically involved in determining osteoblast life span, as it induces apoptosis, inhibits osteoblast proliferation and stimulates osteoblast differentiation through the Akt–FoxOs pathway^286^ to regulate bone remodeling.^287^

The remaining osteoblasts (those on the bone trabecular endosteal and endocortical surfaces) might differentiate into BLCs, which are characterized by lower biosynthetic activity and a flat morphology, or they might remain embedded in the matrix, undergo the active process of osteocytogenesis,^288^ and become osteocytes (5%–20% of mature osteoblasts). Osteocytogenesis requires proteolytic activity to deepen the osteoid. Collagenase-resistant type I collagen in the bone matrix increased the osteocyte apoptosis rate.^289^ Additionally, the deletion of the metalloproteinase MT-MMP1 generated an altered osteocytic network in mice.^290^ Osteocytogenesis requires cytoskeleton plasticity to form dendrites (dendrogenesis). Although the entire dendrogenesis process has not yet been elucidated, one factor known to be necessary in the dendrogenesis of osteoid osteocytes (the late osteoblast/early osteocyte that undergoes the transition to mature osteocytes^288^) is E11/gp38 or podoplanin (Pdpn), a transmembrane glycoprotein. The conditional deletion of PDNP in mice generated a decreased number and length of osteocyte processes.^291^ Recently, it has been shown that SP7 (Osterix) is also necessary to support dendrogenesis because it regulates Osteocrine.^292^

With the progression of osteocytogenesis, osteoid osteocytes start expressing specific cytoskeletal markers (fimbrin and destrin) and factors for mineralizing the surrounding matrix (Phex and MEPE) until they become mineralizing osteocytes (expressing Dmp1). The cell terminally differentiate into mature osteocytes producing SOST and FGF23.^288^

The differentiation of osteoblasts into osteocytes at the end of the bone remodeling process is essential to reestablish mechanical competence. Therefore, 9.1 million osteocytes are replaced daily during bone remodeling.^16^ Hence, osteocytes can be considered dynamic cells capable of reconstituted mechanical sensitivity after replacement^293^ as well as the inhibitory control over osteoclastogenesis *via* the local production of OPG.^77^

## Conclusions: bone remodeling as a continuous flow of information and connected events

Current evidence on bone remodeling has indicated the complexity of this process by showing how events and effectors are connected and coordinated to ensure the removal of old bone and maintain bone mechanical competence. Recent findings have shown that bone remodeling is a physiological process consisting of continuous operations with fast reactive components that confer survival advantage. After BRC formation, local coordinating factors and events, acting in concert in time and space and responding to metabolic and mechanical regulators, affect the final quantum of BMU activity. This complete picture overwrites the classical view of bone remodeling organized in discrete stages. The current knowledge indicates that each BMU component is critical for different mechanisms depending on the specific differentiation stage of the cell. All the signals discussed in the present review affect the remodeling process. The outcome depends on the cells generating the stimulus, and the way in which the stimulus is transferred. Understanding how the events determining the focal balance between bone resorption and formation unfold within each BMU provides necessary biological background information that will help to better identify targets of therapeutic approaches to osteoporosis.
